# Paper-and-pencil versus computerized administration mode: Comparison of data quality and risk behavior prevalence estimates in the European school Survey Project on Alcohol and other Drugs (ESPAD)

**DOI:** 10.1371/journal.pone.0225140

**Published:** 2019-11-20

**Authors:** Emanuela Colasante, Elisa Benedetti, Loredana Fortunato, Marco Scalese, Roberta Potente, Arianna Cutilli, Sabrina Molinaro

**Affiliations:** National Research Council of Italy, Institute of Clinical Physiology, Epidemiology and Health Research Lab, Pisa, Italy; Leibniz Institute for Prevention Research and Epidemiology BIPS, GERMANY

## Abstract

**Purpose:**

The aim of this experimental study was to investigate whether paper-and-pencil and computerized surveys administered in the school setting yield equivalent data quality indicators and risk behavior prevalence estimates.

**Methods:**

Data were drawn from the European School Survey Project on Alcohol and Other Drugs (ESPAD®) carried out in Italy to monitor drug, alcohol, tobacco use and other risk-behaviors among Italian high school students aged 15–19 years.

A sub-sample of schools was recruited for the study (1673 pupils). For each school, two entire randomly selected courses (from the first to the fifth grade) participated and were assigned randomly to the self-administered paper-and-pencil (N = 811) or computerized survey (N = 862). Differences in data quality were assessed using the following indicators: questionnaire completeness (missing gender and/or 50% of missing answers) and internal consistency (repetitive extreme response patterns). Separate logistic regression models were used to estimate the mode effect on the reporting of each risk behavior, controlling for gender and age. Finally, the prevalence estimates of the experimental study were compared to the results of the national ESPAD® study.

**Results:**

The computerized administration mode produced a higher proportion of invalid questionnaires, but the prevalence estimates generated from responses to the paper-and-pencil and computerized surveys were generally equivalent. Nevertheless, comparing these results with those of the national ESPAD® study, some differences in the prevalence rates were found.

**Conclusions:**

The findings suggest that in a proctored school setting, the computerized survey mode yields almost the same results as the paper-and-pencil mode. However, because of the reliance on existing informatics facilities until when all schools in the country will be sufficiently equipped for the computerized data collection, they should be given the opportunity to choose between paper-and-pencil and computerized survey modes, in order to avoid a possible selection bias.

## Introduction

Over the past 20 years, there has been an increasing use of computerized surveys for data collection in health and social science research. The advantages of this kind of administration mode over self-administered paper-and-pencil (P&P) questionnaires include, in particular, the reduced data-collection costs, the avoidance of data entry errors and the accessibility of data in a short time. These advantages are contributing to a greater consideration in the management of cross-sectional studies that use self-administered questionnaires, leading to the progressive replacement of the P&P method with the computerized testing [1,2]. However, the effect of survey mode on respondents’ reporting and prevalence estimates is still under evaluation.

It is well known that data collection methods can affect participants’ answers to sensitive questions (e.g. drug use and sexual behaviors) and that the validity of self-reports may be affected by respondents’ perception of the level of privacy and confidentiality guaranteed [3–8]. In the case of adolescents’ self-reports of sensitive behaviors, although it is generally assumed that self-administration leads to increased disclosure of sensitive and socially undesirable behaviors as compared to interviewer-administered surveys [3,4,9,10], other factors could play a role in misreporting, such as the setting (e.g. the presence of others while responding) and being assured about the anonymity [7].

In the case of computerized surveys, adolescents’ perception of privacy becomes a crucial concern, especially when group administration takes place in a proctored setting, i.e. a supervised setting such as a computer lab at school, where responses are visible on large desktop computer screens. Some studies have shown that a mode effect exists and adolescents are less compliant with computerized testing when desktop computers are positioned close to one another and they believe that others may see their answers [6]. Respondents to computer-assisted self-interviews in the classroom setting perceived less privacy and anonymity of their responses than did P&P respondents [2]. These perceptions are especially critical as they impact respondents’ willingness to disclose socially undesirable behaviors [9, 11]. However, other studies did not find evidence of a difference between P&P and computer-assisted conditions concerning both the perceived privacy and confidentiality and the estimated risk behavior prevalence [8,9,12, 13].

As of data quality, supervised and standardized computer-based versus P&P testing in group settings could result in mode-specific response behaviors, reflecting differences in the level of commitment to the test. Studies comparing the data quality with respect to the number of omitted responses using P&P versus computerized surveys among adolescents produced contrasting results. Some studies found more incomplete questionnaires in the computerized administration [2] whereas, in others, the P&P mode produced more missing data [13, 14,15].

Moreover, concerning the under-reporting of sensitive issues, results from school-based studies comparing prevalence of risk behavior produced by computer- and P&P administered surveys have been inconsistent. Some studies did not find major differences in youth self-disclosure between computerized and P&P questionnaires on both health measures [12,16,17] and sensitive behaviors as long as measures are taken to protect students’ privacy [2, 6, 18, 19]. A mode effect was instead detected by another study concerning a single risk behavior (smoking or alcohol experience), with the P&P mode producing the higher prevalence estimates [20]. In addition, other studies indicate an increased reporting of some sensitive behaviors among adolescents on computer administration compared to P&P techniques [21–24].

Self-administered questionnaires are frequently used in secondary schools to monitor adolescent drug use and related risk behaviors. In Italy, since 1999 the National Research Council has been conducting a nationally representative survey among high school students to collect data on alcohol, tobacco, drugs and other risk behaviors (European school Survey Project on Alcohol and other Drugs—ESPAD®) in the sampled schools during a regular school day. The survey has been administered since its inception with a paper-and-pencil questionnaire following the European ESPAD standardized methodology [25–26]. Switching to a computerized survey would require all the sampled schools to have appropriate informatics facilities (including computer laboratories, a sufficient number of computers and a working Internet connection) to survey multiple classes of students in a short timeframe. Therefore, before making any changes in the mode of administration for an ongoing European and national surveillance system, it is critical to consider the potential effects that such changes might have on the data quality and prevalence estimates generated from responses [2,4]. These assessments are particularly needed since such changes could disrupt the comparability of results and the ability to assess trends over time [19].

The purpose of this experimental study conducted in the framework of the ESPAD survey on a sub-sample of Italian high school students was to investigate the comparability and quality of survey data obtained via computerized administration mode versus those obtained via P&P questionnaire. With this aim, the survey was administered as a computerized self-administered questionnaire in a standardized proctored setting (a test administrator supervised the whole testing procedure and remained present during test taking in the classroom) under the same conditions as the paper version, in order to investigate mode differences (e.g., paper-based vs. computer based) concerning both data quality and prevalence estimates generated from responses. The specific aspects considered to assess data quality are the questionnaire completeness (missing gender and/or missing answers to more than 50% of questions) and the internal consistency (repetitive extreme response patterns). The prevalence estimates analysed concern a wide range of sensitive behaviors (e.g. substance use, including the consumption of alcohol, tobacco, and illicit drugs). The estimates produced by the experimental study were then compared to those of the national ESPAD® study.

## Materials and methods

### Sample and procedure

The present study was conducted in Italy in the framework of the ESPAD study. ESPAD is a European cross-national survey conducted every four years in more than forty European countries with the aim to collect comparable data on substance use among high school students aged 16 under the same standardized conditions.

The ESPAD methodological protocol, established in the early 1990s, includes a master questionnaire and a standardized procedure for the survey administration in the sampled schools. The pupils fill in the ESPAD questionnaire in the classroom setting, supervised by a survey leader (a teacher or research associate). P&P is the typical data collection mode, although few countries have been allowed to perform computerized data collections.

The last European data collection wave was conducted in 2015. A full description of the sampling and data collection procedure, as well as of the data cleaning rules, is reported in the 2015 ESPAD Report [25] and in the ESPAD 2015 Methodology report [26].

In Italy, the ESPAD® study is conducted every year by the National Research Council to monitor drug, alcohol, tobacco use, and other risk-behaviors among Italian high school students aged 15–19 years using the same methodological protocol as the European study. Every four years, data about the Italian pupils aged 16 are provided to the European study and merged into the international ESPAD database.

The Italian ESPAD® study received the ethical review by the Research Ethics and Bioethics Committee of the National Research Council. Concerning the consent procedures, passive parental consent is used. Specific information letters are provided to participating schools, parents/guardians, teachers and pupils illustrating the aims of the project, the procedure of administration of the survey, including all the measures taken to ensure the privacy and anonymity of participants (pupils are requested not to include their name or any other information which could identify them), as well as the dissemination of results. The participation is voluntary and pupils can decide not to take part or to withdraw at any time. The results of the study are published only at aggregate level, no data are presented by single class or school.

For the present study, a sub-sample of 79 schools was selected respecting the national distribution in terms of geographical location (North, Centre, South and islands) and type of school (lyceums, art institutes, vocational institutes). Those selected schools that had the informatics facilities to accommodate the computerized administration of the questionnaire to two courses of pupils and that were willing to participate were recruited for the study. The schools refusing to participate were replaced with other randomly drawn schools sharing the same characteristics (geographical location and type).

Out of the final sample of 79 schools that were recruited for this comparison study, 25 did not follow-up to the formal acceptance due to different reasons intervened following the agreement to participate, and specifically: five were not able to reserve the computer lab to the computerized administration for a whole student course (i.e. five classes), three did not have anymore two courses (from the first to the fifth grade each) available to be involved in the study due to unforeseen extra activities, five refused due to the later involvement in other compulsory projects, 12 without specific reasons. Of the remaining 54 schools that were provided with the necessary equipment to participate in the study, 11 did not return the materials, four performed only the computerized administration, and 27 only the P&P administration (Table 1). These schools where therefore excluded from the study.

**Table 1:** Geographical distribution of Italian school’s sample.

National Distribution ESPAD®Italy 2015		Total sub- sample		Computerized and P&P administration		Not returned	Only P&P	Only computerized
Area	%	n = 54	%	n = 12	%	n = 11	n = 27	n = 4
North	38	19	35	3	25	5	10	1
Centre	18	10	19	2	17	3	4	1
South	44	25	46	7	58	3	13	2

A final sample of 12 schools actually participated in the comparison study. In each school, two complete courses (a course includes five classes, from the first to the fifth grade) were randomly drawn to participate in the study, for a total of 1673 pupils interviewed. The selected classes within each school were assigned randomly either to P&P (N = 811) or computerized (N = 862) condition.

### Measures and data quality

The ESPAD® questionnaire collects information about licit and illicit drugs in terms of prevalence of use (lifetime, last year and last month), and additional questions about leisure activities, relationships at school, attitude concerning drug use, satisfaction with relationships with parents or friends, social and cultural status.

Sensitive questions used in this study are related to the use of tobacco and alcohol, alcohol intoxication, binge drinking, use of energy drinks, tranquillizers or sedatives without medical prescription, anabolic steroids, cannabis and any other drugs use (ecstasy, amphetamine, methamphetamine, cocaine, crack, inhalants, LSD, heroin, magic mushroom, GHB). Furthermore, an overall effect across all sensitive questions was considered (use of cigarette, alcohol or any illicit drug).

Differences in data quality between P&P and computerized administration mode were assessed using three indicators developed in the 2015 ESPAD® survey, following the ESPAD guidelines for data cleaning: questionnaires with a missing answer for gender; questionnaires with missing answers to 50% of the questions composing the core questionnaire (i.e. the set of questions that in the European questionnaire are compulsory in every participating country) and percentage of records where the respondent appeared to have followed patterns involving repetitive marking of extreme values, i.e. maximum use of all substances (more than 40 times). The data cleaning process followed the ESPAD® guidelines: questionnaires presenting a missing answer for gender, missing answers to more than 50% of the core questions and/or patterns involving repetitive marking of extreme values were therefore deleted [25, 26].

### Data collection procedure

#### P&P questionnaire and OCR acquisition

The paper-and-pencil administration followed the standard procedures foreseen by the ESPAD study [25–26]. The questionnaire was administered to pupils in their regular classroom. Pupils recorded their responses in a computer-scannable questionnaire booklet and were instructed to seal their completed questionnaire in a blank envelope before inserting it into a box common to the whole class provided by the data collector.

The P&P questionnaire was implemented taking into account the use of Optical Character Recognition (OCR) technologies which, through an appropriate data conversion, allow to automatically store hand-written survey forms. The stages of the OCR process involve the questionnaires scanning, the interpretation of the recognized information, the checking of the goodness of data, and the production of the final data output into an electronic format compliant with standard statistical tools. If any ambiguity is found during the data acquisition process, the scanning software highlights non-recognizable characters and stops for a manual check by the operator. Once all steps are completed, the data are ready for further cleaning process.

#### Computerized condition and web platform

The electronic questionnaire was administered in the school’s stationary computer laboratory. After introducing the survey and directing pupils to the website where the questionnaire was hosted, data collectors gave a unique password to the whole class. Pupils logged into the questionnaire using the class password. Up to 6 questions appeared on each screen and response choices remained visible until pupils clicked the ‘‘next” button on the bottom of each screen. To enhance comparability with the paper-and-pencil condition, web pages were structured in a similar way to paper pages, with the same number of questions per page and the same content. No warning messages in case of missing or inconsistent values were included. Moreover, despite skip patterns are being widely used in computerized surveys, respondents were allowed to proceed to subsequent questions regardless of whether they left some questions in blank. The technological environments chosen for the implementation of the Web ESPAD® platform are completely open-source (MySQL database, PHP development language, LimeSurvey application). Once the survey is completed, the obtained data are available in electronic format, ready for further cleaning process.

### Statistical analysis

The percentage of missing answers for gender, as well as the percentage of missing answers to 50% of the core questions, maximum use of all substances and percentage of deleted questionnaires were calculated for both P&P and computerized questionnaires. Differences were detected by Chi-square test.

Chi-square analyses examined differences in pupils’ demographic characteristics (gender and age) by questionnaire mode, and results were considered significant at p < 0.05.

Prevalence of pupils reporting engaging in several risk behaviors were calculated by mode.

Separate logistic regression models were used to estimate the mode effect on the reporting of each risk behavior. Wald F p-value < 0.05 was considered statistically significant. Each model was controlled for gender and age to avoid possible confounding effect.

Adjusted Odds Ratios (OR) and 95% Confidence Interval (CI) were calculated for the estimates of mode effect on each prevalence (P&P administration was adopted as reference).

Furthermore, each prevalence was compared with the prevalence detected in the national 2015 ESPAD® survey, conducted using the P&P method, using two sample proportion test.

All analyses were conducted using the STATA statistical package, version 10.

## Results

All the selected data quality indicators varied significantly by questionnaire mode. The computer-assisted mode generated a significantly higher proportion of missing answers on gender, cases with less than 50% of the core questions answered and cases with repetitive extreme response patterns. Globally, the computer-assisted mode produced a higher proportion of deleted questionnaire (Table 2).

**Table 2:** Data quality and deleted cases, percentages by mode.

ESPAD®Italy 2015 Comparison study (P&P vs computerized administration mode)		P&P	Computerized	Pearson	p-value
		N = 811	N = 862	chi2 (df)	
Cases with missing on gender (%)		1.7	4.9	12.8 (1)	0.00
Cases with less than 50% of the core questions answered (%)		0.5	7.2	49.5 (1)	0.00
Cases with repetitive extreme response patterns (%)		1.2	2.9	5.7 (1)	0.02
Total deleted cases	N	27.0	91.0		
	%	3.3	10.6	33.3 (1)	0.00
Valid cases		784	771		

No significant differences were instead detected concerning pupils’ demographic characteristics (Table 3).

**Table 3:** Demographic characteristics of students in the study sample, overall percentages, and percentages by mode.

ESPAD®Italy 2015: Comparison study (P&P vs computerized administration mode)					
	Sample (%) N = 1555	P&P (%) N = 784	Computerized (%) N = 771	Pearson chi2 (df)	p-value
Gender					
Male	59.0	61.4	56.5	3.7 (1)	0.06
Female	41.0	38.6	43.5		
Age (years)					
15	17.9	17.6	18.2	2.9 (4)	0.58
16	23.4	24.0	22.8		
17	22.0	21.3	22.7		
18	18.9	18.0	19.8		
19	17.8	19.1	16.5		

Table 4 reports the prevalence of each risk behavior by mode and the related adjusted OR. Mode was associated significantly with the reporting of just one risk behavior, i.e. last year alcohol use, and the odds of reporting this behavior among respondents to the P&P was higher than among respondents to the computerized survey.

**Table 4:** Prevalence of students reporting engaging in risk behaviors by mode and Adjusted Odds Ratios (OR).

ESPAD®Italy 2015: Comparison study (P&P vs computerized administration mode)							
Sensitive behaviors			P&P vs Computerized (P&P is referent)				
	P&P (%)	Computerized (%)	Adjusted OR*	CI 95%		Wald (df)	p-value
	N = 784	N = 771					
Lifetime cigarette use	59.3	57.8	0.95	0.79	1.14	0.3 (1)	0.62
Lifetime alcohol use	88.4	84.1	0.80	0.65	1.00	3.9 (1)	0.05
Last year alcohol use	82.1	76.3	0.78	0.64	0.96	5.5 (1)	0.02
Last month alcohol use	62.3	58.0	0.87	0.72	1.05	2.1 (1)	0.15
Binge drinking during last month	36.0	39.6	1.18	0.96	1.44	2.5 (1)	0.11
Lifetime intoxication from drinking alcohol	45.4	41.2	0.86	0.71	1.04	2.5 (1)	0.14
Last year intoxication from drinking alcohol	34.3	31.1	0.87	0.71	1.07	1.7 (1)	0.19
Last month intoxication from drinking alcohol	15.5	14.2	0.89	0.67	1.19	0.5 (1)	0.46
Lifetime cannabis use	34.5	31.2	0.88	0.71	1.09	1.3 (1)	0.25
Last year cannabis use	28.2	26.1	0.92	0.74	1.16	0.5 (1)	0.49
Last month cannabis use	18.3	16.4	0.89	0.68	1.17	0.7 (1)	0.39
Lifetime ecstasy use	2.8	2.3	0.89	0.47	1.68	0.1 (1)	0.72
Last year ecstasy use	2.4	1.7	0.73	0.35	1.49	0.7 (1)	0.39
Lifetime amphetamines use	1.9	1.8	1.00	0.48	2.09	0.0 (1)	0.99
Last year amphetamines use	1.7	1.6	1.00	0.45	2.23	0.0 (1)	0.98
Lifetime methamphetamines use	2.2	1.5	0.71	0.34	1.53	0.8 (1)	0.39
Last year methamphetamines use	1.8	1.2	0.70	0.30	1.63	0.7 (1)	0.40
Lifetime cocaine use	3.1	2.6	0.90	0.49	1.65	0.1 (1)	0.73
Last year cocaine use	2.6	2.4	0.99	0.52	1.90	0.0 (1)	0.98
Lifetime crack use	2.4	1.3	0.57	0.26	1.22	2.1 (1)	0.15
Last year crack use	2.2	0.9	0.44	0.18	1.07	3.3 (1)	0.07
Lifetime inhalants use	3.1	2.9	0.98	0.54	1.77	0.0 (1)	0.94
Last year inhalants use	1.8	1.2	0.70	0.30	1.63	0.7 (1)	0.40
Last month inhalants use	1.5	0.8	0.55	0.21	1.49	1.4 (1)	0.24
Lifetime tranquillizers or sedatives (without a doctor’s prescription) use	4.7	5.1	1.05	0.66	1.66	0.0 (1)	0.85
Lifetime LSD or some other hallucinogens use	3.1	2.0	0.67	0.35	1.28	1.5 (1)	0.22
Lifetime heroin use	1.4	1.2	0.88	0.36	2.14	0.1 (1)	0.78
Lifetime magic mushrooms use	3.1	1.8	0.64	0.33	1.25	1.7 (1)	0.19
Lifetime GHB use	1.1	0.5	0.50	0.15	1.64	1.3 (1)	0.26
Lifetime anabolic steroids use	2.0	1.3	0.68	0.31	1.52	0.9 (1)	0.35
Lifetime use of alcohol together with pills (medicaments) in order to get high	3.3	2.6	0.79	0.44	1.43	0.6 (1)	0.44
Lifetime energy drink use	56.3	55.6	1.02	0.83	1.25	0.0 (1)	0.85
Last year energy drink use	43.0	39.8	0.92	0.74	1.14	0.5 (1)	0.46
Last month energy drink use	28.7	27.2	0.98	0.77	1.24	0.0 (1)	0.84
Lifetime use of energy drinks and alcohol during a single session	34.4	34.1	1.03	0.83	1.29	0.1 (1)	0.76
Last year use of energy drinks and alcohol during a single session	27.8	26.2	0.97	0.76	1.23	0.1 (1)	0.77
Last month use of energy drinks and alcohol during a single session	17.2	17.0	1.04	0.79	1.37	0.1 (1)	0.81
Lifetime use of cigarette, alcohol or any illicit drug	92.5	90.0	0.82	0.63	1.05	2.5 (1)	0.11

* Adjusted for gender and age

As shown in Table 5, comparing both P&P and computerized administration with the national ESPAD® study, statistically significant differences between national and P&P prevalence were found for lifetime alcohol intoxication and lifetime and last year use of both energy drinks and alcohol during a single session. Differences between the national prevalence and the prevalence generated from responses to the computerized survey were found for lifetime and last month alcohol use, binge drinking during the last month and lifetime use of both energy drinks and alcohol during a single session.

**Table 5:** Comparison with the National ESPAD®Italy 2015 study.

Risk behaviors				Two-sample proportion test			
	National (%)	P&P (%)	Computerized (%)	National vs P&P		National vs Computerized	
	N = 17257	N = 784	N = 771	z	p-value	z	p-value
Lifetime cigarette use	60.8	59.3	57.8	0.83	0.41	1.69	0.09
Lifetime alcohol use	87.0	88.4	84.1	-1.17	0.24	2.30	0.02
Last year alcohol use	79.2	82.1	76.3	-1.95	0.05	1.93	0.05
Last month alcohol use	61.8	62.3	58.0	-0.27	0.79	2.13	0.03
Binge drinking during last month	35.5	36.0	39.6	-0.31	0.76	-2.35	0.02
Lifetime intoxication from drinking alcohol	41.7	45.4	41.2	-2.01	0.04	0.33	0.74
Last year intoxication from drinking alcohol	31.1	34.3	31.1	-1.89	0.06	-0.05	0.96
Last month intoxication from drinking alcohol	13.6	15.5	14.2	-1.54	0.12	-0.46	0.64
Lifetime cannabis use	32.5	34.5	31.2	-1.19	0.24	0.72	0.47
Last year cannabis use	26.6	28.2	26.1	-0.95	0.34	0.29	0.77
Last month cannabis use	17.4	18.3	16.4	-0.71	0.48	0.70	0.49
Lifetime ecstasy use	2.8	2.8	2.3	-0.05	0.96	0.73	0.47
Last year ecstasy use	2.2	2.4	1.7	-0.38	0.70	1.00	0.32
Lifetime amphetamines use	2.0	1.9	1.8	0.19	0.85	0.37	0.71
Last year amphetamines use	1.6	1.7	1.6	-0.06	0.95	0.14	0.88
Lifetime methamphetamines use	1.8	2.2	1.5	-0.77	0.44	0.69	0.49
Last year methamphetamines use	1.5	1.8	1.2	-0.76	0.45	0.61	0.54
Lifetime cocaine use	3.7	3.1	2.6	0.95	0.34	1.59	0.11
Last year cocaine use	2.7	2.6	2.4	0.31	0.76	0.64	0.52
Lifetime crack use	2.3	2.4	1.3	-0.18	0.86	1.83	0.07
Last year crack use	1.6	2.2	0.9	-1.19	0.23	1.51	0.13
Lifetime inhalants use	2.7	3.1	2.9	-0.59	0.55	-0.26	0.79
Last year inhalants use	1.7	1.8	1.2	-0.29	0.77	1.02	0.31
Last month inhalants use	1.3	1.5	0.8	-0.45	0.65	1.33	0.18
Lifetime tranquillizers or sedatives (without a doctor’s prescription) use	5.3	4.7	5.1	0.73	0.47	0.25	0.80
Lifetime LSD or some other hallucinogens use	2.9	3.1	2.0	-0.35	0.72	1.45	0.15
Lifetime heroin use	1.8	1.4	1.2	0.76	0.45	1.20	0.23
Lifetime magic mushrooms use	2.7	3.1	1.8	-0.70	0.49	1.38	0.17
Lifetime GHB use	1.1	1.1	0.5	-6.00	0.95	1.52	0.13
Lifetime anabolic steroids use	1.5	2.0	1.3	-1.36	0.17	0.26	0.79
Lifetime use of alcohol together with pills (medicaments) in order to get high	3.3	3.3	2.6	-0.03	0.97	1.03	0.30
Lifetime energy drink use	56.8	56.3	55.6	0.31	0.75	0.67	0.50
Last year energy drink use	42.1	43.0	39.8	-0.51	0.60	1.20	0.23
Last month energy drink use	28.4	28.7	27.2	-0.20	0.84	0.66	0.51
Lifetime use of energy drinks and alcohol during a single session	29.8	34.4	34.1	-2.72	0.01	-2.47	0.01
Last year use of energy drinks and alcohol during a single session	23.5	27.8	26.2	-2.76	0.01	-1.69	0.09
Last month use of energy drinks and alcohol during a single session	15.6	17.2	17.0	-1.18	0.24	-1.02	0.31
Lifetime use of cigarette, alcohol or any illicit drug	91.8	92.5	90.0	-0.66	0.51	1.60	0.11

## Discussion and conclusions

School surveys on risk behaviors have been traditionally performed using the P&P administration mode, although computer-based surveys are increasingly used. This study was the first carried out among a large representative sample of Italian high school students, using a standardized questionnaire [27] validated in the framework of the ESPAD cross-national research project, aimed at comparing the reporting of risk behaviors between the P&P and computerized administration mode. Regarding the data quality, the P&P mode showed better results as respondents were more likely to provide a response to the gender question and to answer to more than 50% of the core questions. Furthermore, the P&P administration produced less cases with repetitive extreme response patterns than the computerized survey. These findings are similar to those of other studies that found more incomplete questionnaires and less valid responses in the computer-assisted condition [2,8]. The lower data quality produced by the computerized administration could be a consequence of the fact that, although having started to complete the questionnaire, some students might have had a higher change to multitask (the computer must be connected to the Internet and, although not allowed, it is possible to have multiple Internet pages open at the same time while completing the survey). Therefore, they might have focused less on completing the questionnaire leading to higher levels of item non-response, drop out and repetitive extreme response patterns.

In our study, respondents did not differ significantly by administration condition for gender or age.

Regarding the prevalence estimates generated from responses, our comparison study suggests that in a supervised school setting, under equivalent conditions, there is no mode effect in the reporting of sensitive information between P&P and computerized administration mode: no statistically significant differences were found either for self-reported prevalence or for the overall effect, except for self-reported last year alcohol use, with a statistical significance between 0.01 and 0.05. These findings are in line with previous studies reporting no or very few differences between P&P and computerized surveys [14,16,20,28–32], whilst do not confirm those of other studies that found a higher adolescents’ self-disclosure of sensitive behaviors in computerized surveys [21–23,33]. On the other side, some differences between the prevalence estimates generated by the comparison study and those of the national ESPAD® study were detected. The possibility to accommodate the computerized administration of the survey depends on the availability in schools of both informatics facilities and a stable Internet connection. The challenge of switching to the computerized survey is also associated with the difficulty of scheduling the data collection, because computer laboratories are often not sufficient for accommodating both the curricular and extra-curricular activities of all student classes and need to be reserved months in advance. Although the access to computer labs and Internet connection should be universally guaranteed in Italian schools, the results of our study suggest that this is not always the case. When randomly selected, some schools refused to participate because they lacked the necessary informatics facilities. Some others, despite having agreed to participate and although disposing of a computer laboratory, administered only the P&P questionnaire because they could not reserve the computer lab for the survey due to the execution of other compulsory curricular activities. Furthermore, participating schools were more likely to be from a specific geographical location (i.e. South of Italy). This could result in a possible self-selection bias of the sample: schools that have sufficient informatics facilities to accommodate the computerized administration of the questionnaire to whole classes of pupils and that are willing to participate are recruited for the study, whereas schools that do not have it are excluded from the sample.

In conclusion, our study provides further evidence that in a proctored school setting, the computerized administration of a survey yields almost the same results as paper-and-pencil administration.

Computerized surveys are definitively a suitable alternative to P&P surveys due to the reduced costs and time needed for the data collection. However, the transition to this administration mode requires conditions related to the availability of informatics facilities that seem far from being met by the current Italian high school system. Until when all high schools in the country will be sufficiently equipped, researcher should give the opportunity to the selected school to choose between the two types of administration in order to avoid a possible selection bias.

The main limitation of the present study is the restricted number of schools that succeeded in completing both the P&P and the computerized administration, generating a limited sample size with respect to the one initially planned. However, this constitutes a finding on its own, since it suggests that a completely computerized survey would not be currently sustainable in Italy. One of the consequences of this limitation is that, since some risk behaviors have a very low prevalence, the limited sample size makes it difficult to identify a significant difference between administration modes and in the comparison with the national sample.

Future developments of this research might take into consideration also other interesting socio-demographic characteristics, for example the type of school, as well as sensitive behaviors (for example some aspects of the relationship with parents). This could definitely deepen the knowledge of the factors associated with the different levels of participation of the schools and provide further insights into the effects of the survey administration mode on adolescent respondents.

Since the prevalence estimates generated by the responses to the computerized survey appear overall comparable to those produced by the P&P survey, there might be solutions to potentially overcome the two issues identified with using the computer-assisted mode (lower data quality and a potential selection bias).

In fact, the lower data quality could be partly solved by including in the computerized survey some features, like for example soft warnings in case of missing values or extreme answers. However, the introduction of these elements would need to be carefully assessed as these do not prevent respondents from providing inexact answers in order to quickly proceed through the survey, and might compromise the comparability with the results of the previous P&P data collections. The potential selection bias of schools could instead be addressed by equipping them with tablets and Internet access for the ESPAD study. Unfortunately, although this measure would eliminate the costs associated with the provision of the P&P materials and with the data entry procedures, given the large sample size of the Italian ESPAD study, the financial resources needed to adopt them would be far higher and, at the present stage, not sustainable.

## Supporting information

S1 File (XLSX)

S2 File (XLSX)
